# A Systematic Review of the Effects of Low-Carbohydrate Diet on Athletic Physical Performance Parameters

**DOI:** 10.7759/cureus.79166

**Published:** 2025-02-17

**Authors:** Zeeshaan H Sultan, Diana Speelman

**Affiliations:** 1 Biochemistry, Lake Erie College of Osteopathic Medicine, Erie, USA

**Keywords:** aerobic performance, athletic performance, ketogenic diet, low-carbohydrate high-fat diet, lower body strength, strength

## Abstract

A low-carbohydrate diet (LCD) or ketogenic diet is commonly used by individuals who want to achieve ketosis, which can boost fat metabolism, increase insulin sensitivity, and reduce blood sugar spikes. However, many athletes are hesitant to utilize a diet that specifically restricts the intake of carbohydrates, an important source of energy for physical activity. Athletes usually adopt a high-carbohydrate or high-protein diet, depending on their goals. This review aims to examine the evidence for the impact of an LCD on athletes’ aerobic, anaerobic, and upper and lower body strength performance. A systematic review of original studies was conducted for articles indexed in PubMed that utilized an LCD in athletes and reported athletic performance values. A total of 19 studies were included in the final synthesis. An LCD may aid in maintaining or increasing upper and lower body strength. However, this type of diet provides no consistent benefit and may even negatively impact some measures of aerobic and anaerobic performance. Taken together, no strong evidence indicates the benefit of an LCD on athletic performance. Athletes wishing to modify their diet for improved performance should consider possible diets with specific training and performance goals in mind.

## Introduction and background

Diet and athletic performance

Diet is an important consideration in athletic performance. Having a consistent, well-balanced diet as an athlete has been shown to correlate with increased success in sports performance [1,2]. Currently, there are a variety of diets to consider, ranging from a vegan diet, ketogenic diet, high-protein diet, intermittent fasting, etc., all of which have attained mainstream popularity. Perhaps the most important dietary consideration for an athlete is the macronutrient ratio, which influences physical performance in anaerobic exercise, aerobic exercise, muscle strength gains, and post-exercise recovery [3].

Carbohydrate-rich diets help athletes replenish muscle glycogen stores, utilized during high-energy demand activities to rapidly produce adenosine triphosphate (ATP) for muscle contraction [4]. Furthermore, carbohydrates are known to help with post-exercise recovery in athletes via the proposed accelerated replenishment of both liver and muscle glycogen [5,6]. A high-carbohydrate diet (75-80% calories from carbohydrates) has been shown to correlate with increased aerobic performance during time-to-exhaustion trials and peak performance power on cycle ergometry testing [7]. Carbohydrates are an essential macronutrient in athletes’ diets to ensure maximum energy availability for muscle contraction [8].

Protein is another essential macronutrient for athletes, supporting gains in muscle mass and strength when combined with strength training to promote hypertrophy of muscle fibers [9]. Dietary protein is also important for recovery from intense exercise by mitigating muscle soreness, helping repair damaged muscle tissue, and enhancing the ability to perform subsequent exercise sessions [10]. High-protein diets for athletes may help improve body composition, with decreased total body mass and body fat percentage [11]. Such diets allow athletes to gain muscle mass and strength, as shown through improvements in maximum strength tests such as one-repetition maximum bench presses, squats, and pull-ups.

Other diets with a slightly higher fat content, like the Mediterranean diet that emphasizes the intake of fish, nuts, fruits, and vegetables, may also provide some benefits, such as increased aerobic performance in athletes in a five-kilometer trial [12]. Furthermore, the Mediterranean diet may counteract muscle strength loss, as evidenced by a study that utilized the Mediterranean diet and showed no significant decrease in maximum squat jump, countermovement jump, hand grip strength, and knee extension maximum velocity contraction in participants who had a year minimum of training experience [13]. As different diets have been shown to have different effects on athletic performance parameters, it is important to analyze each to better help athletes achieve their specific goals.

Rationale for systematic review

Athletes focus on the carbohydrate and protein macronutrient dietary composition rather than fats, as fats do not play direct roles in glycogen storage or muscle hypertrophy. A low-carbohydrate diet (LCD) may help individuals decrease body fat and lose weight by greatly limiting carbohydrates (to <10% caloric intake or <50 g carbohydrates daily) and increasing fat intake; this may also be referred to as a ketogenic diet as it induces a state of ketosis [14]. However, this diet will greatly restrict the two most important macronutrients for athletes and replace them with fats, which may be detrimental to athletic performance. Furthermore, competitive athletes often do not aim for weight loss, as many already have a low body fat percentage. Male athletes have a body fat percentage as low as 5% for bodybuilders. In comparison, female athletes have body fat percentages as low as 10% in elite runners, such that the ketogenic diet is unappealing to a variety of athletes [15,16]. In addition, athletes utilizing a high-fat diet can be at risk of increased total cholesterol levels [17]. Studies on the general population also link high-fat diets with increased risk of diabetes and cardiovascular disease [18]. However, other studies have recently suggested that a low-carbohydrate or ketogenic diet may improve athletes' aerobic, anaerobic, and body strength performance, even compared to a normal high-carbohydrate diet many athletes utilize [19-21].

Objectives of review

The primary goal of this systematic review was to evaluate the experimental designs and results from studies of an LCD on athletic performance. We summarize the participants' demographics, types and length of dietary interventions utilized within each study, athletic performance outcomes measured, dietary adherence measurements, and study limitations. Gaps in knowledge and future directions are also highlighted. This information assesses recommendations for athletes who want to utilize an LCD.

## Review

Literature search

**Figure 1:**
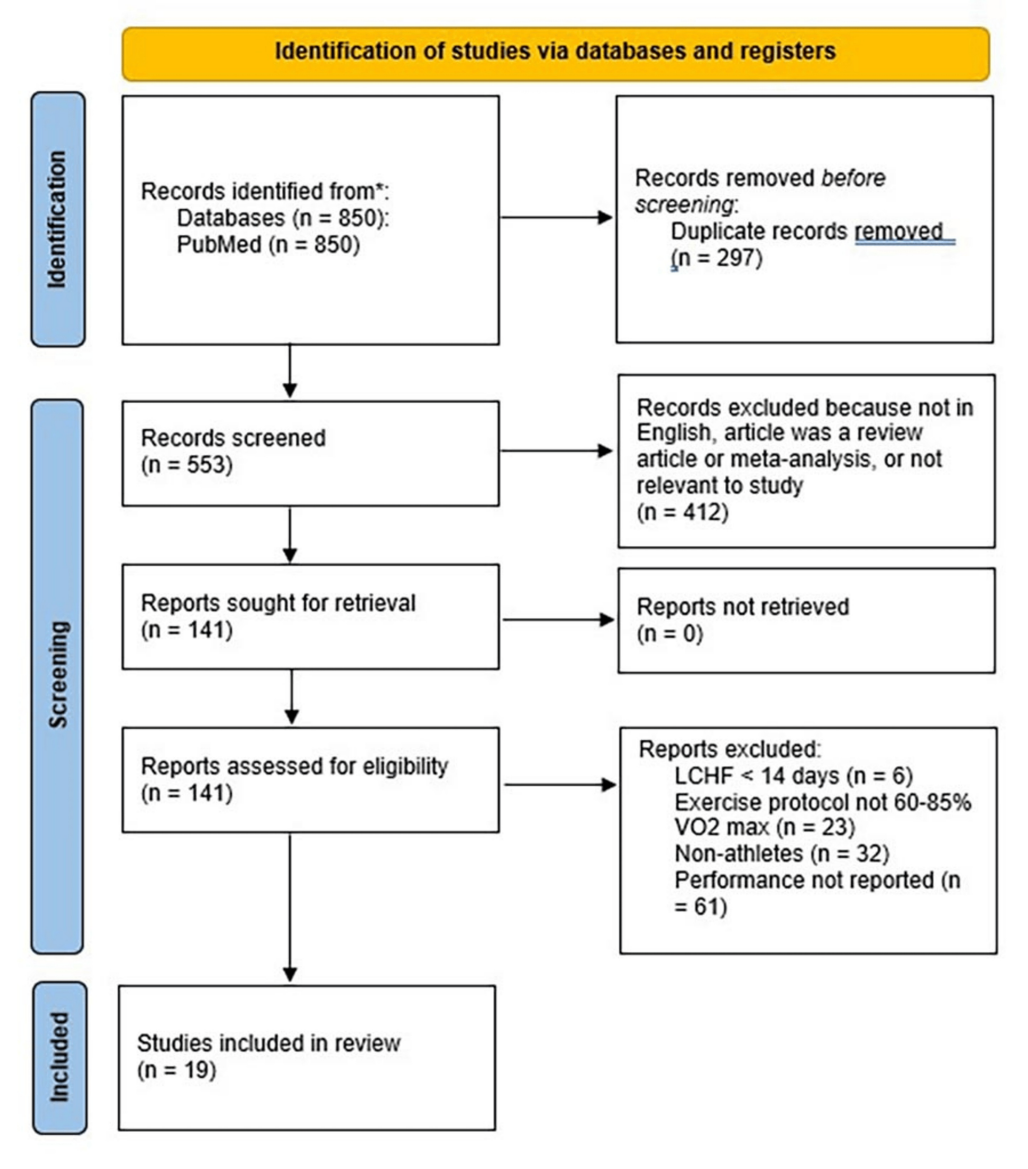
PRISMA flow diagram Process for the literature search, eligibility, and selection for including studies in the systematic review. PRISMA: Preferred Reporting Items for Systematic Reviews and Meta-Analyses, LCHF: low-carbohydrate, high-fat

This systematic review was carried out in accordance with the Preferred Reporting Items for Systematic Reviews and Meta-Analyses (PRISMA), which is summarized in Figure 1. A comprehensive literature search was conducted using the PubMed database from 12 March 2024 to 26 March 2024 for articles published in the last 35 years. The search strategy included an exercise-related term: “athlete” or “endurance performance” or “bicycle” or “swim” or “run”, paired with a ketogenic diet-focused term: “low-carbohydrate” or “LCHF” or “ketogenic”. In total, there were 15 separate searches, totaling 850 results. A list of all articles returned by the searches was made, and all duplicates and non-English language articles were removed from this list.

Eligibility criteria, article screening, and exclusion criteria

One study investigator independently reviewed the titles and abstracts retrieved from the initial literature search based on the relevance to the systematic review objectives. Review articles and meta-analyses were removed, leaving randomized controlled trials and clinical trials. Full-text articles were then screened for those that included a quantitative physical activity measurement in trained individuals or athletes, including pre- and post-ketogenic diet measurements or a comparison of measurements between groups that followed a control or low-carbohydrate, ketogenic diet. The dietary composition needed to be clearly defined for study inclusion, indicating the percentage of calories from macronutrients in the daily diet; some studies also reported carbohydrates in grams per day. Articles excluded were studies that did not report any quantitative physical performance measurements, utilized a low-carbohydrate but high-protein diet, analyzed samples from non-athletes or individuals who were untrained, gave the participants a low-carbohydrate beverage instead of a long-term diet intervention, and compared low-carbohydrate and high-carbohydrate diets without achieving ketosis.

Classification of articles and qualitative synthesis

Articles were classified according to the type of exercise performance tests to synthesize information: anaerobic exercise, aerobic exercise (60-75% VO_2_max), upper extremity strength training, lower extremity strength training, and high-intensity low-volume exercises. For the purpose of this review, aerobic performance was defined as testing parameters of longer, rhythmic movements, such as running 10 kilometers, while anaerobic performance was defined as testing of high-intensity, short bursts of physical activity, such as maximum cycling power in 30 seconds. Studies included randomized controlled trials, clinical trials, and controlled clinical trials.

Data collection

Full-text articles meeting the eligibility criteria for a qualitative synthesis review were reviewed, and one investigator extracted data independently. The extracted data included participant athletic history and demographic information, the number of participants in the final data analysis, the procedure for participant assignment to a group, the macronutrient composition of diets used in the study, the duration of dietary intervention prior to performance testing, a protocol for performance tests (type, frequency, duration), monitoring for dietary adherence, and blood values reported pre- and post-intervention.

Risk of bias

**Figure 2:**
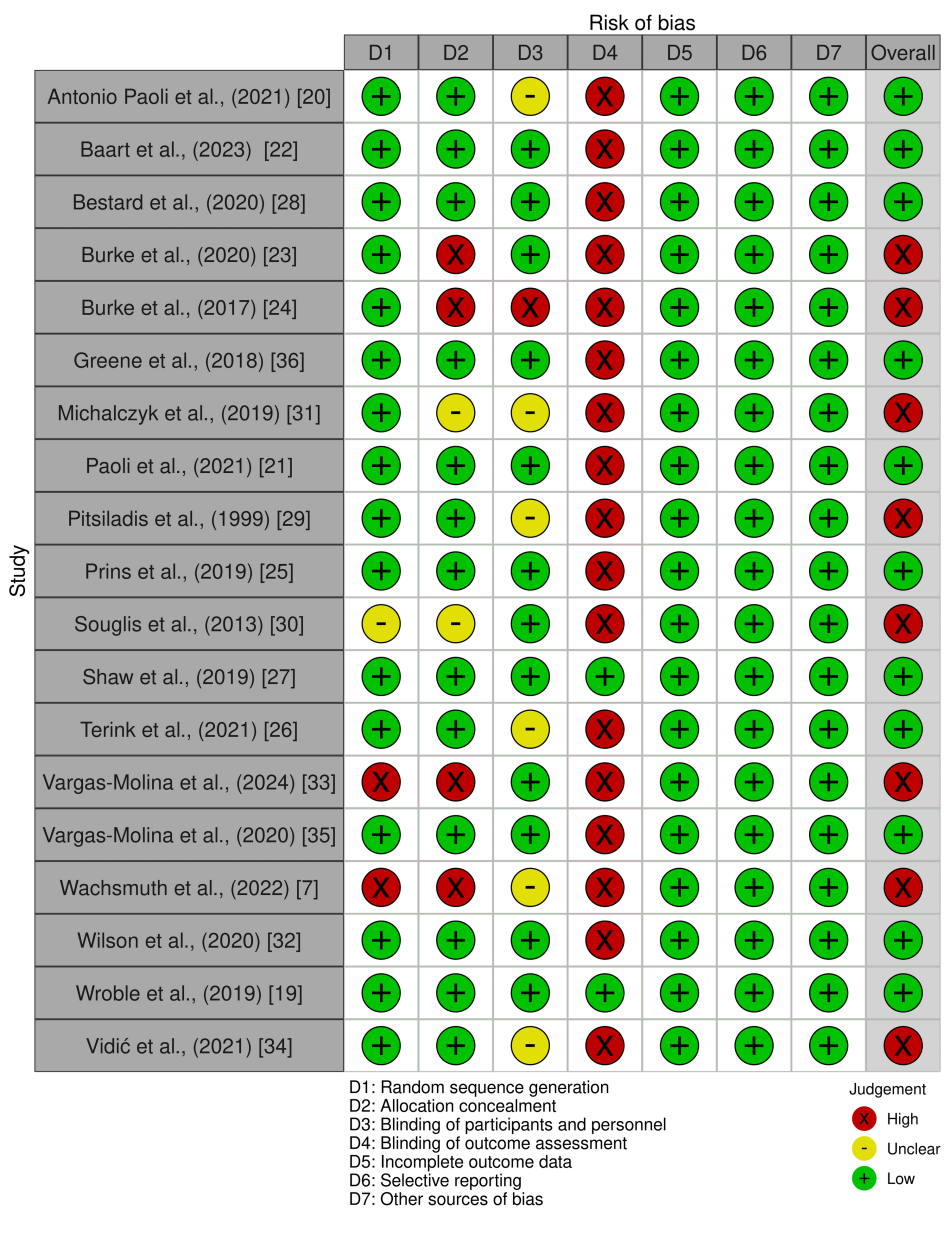
Risk of bias in studies included in the analysis Studies included in the systematic review were assessed for risk of bias across seven domains. An overall risk of bias for each study is shown in the last column.

The risk of bias in individual studies was classified as performance bias (e.g., insufficient control of variables outside of the study protocol), attrition bias, and reporting bias (e.g., not reporting data with non-significant differences). These biases were used to evaluate the strength of the reported data and analyze the effects of a low-carbohydrate, ketogenic diet on athletic performance (Figure 2). All articles, regardless of the results or conclusion of the study, were included in the qualitative synthesis review to avoid selection bias across studies.

Results by type of athletic activity

Aerobic Exercise Performance

Studies of aerobic exercise performance tests for participants on ketogenic diets of varying lengths are summarized in Table 1. In a randomized controlled trial with 16 semi-professional soccer players that followed a 30-day LCD, the Yo-Yo intermittent recovery test (of maximum aerobic fitness) indicated significantly increased aerobic performance on day 30 compared to the pre-dietary intervention baseline [20]. However, a randomized sequence crossover trial with 14 recreational male athletes showed no change in their exercise efficiency or economy after a 14-day LCD [22]. Furthermore, there were no significant changes in 10-km race times for 21 professional male walkers after a 21-day LCD clinical trial [23]. However, a repeat of this clinical trial with 26 professional male and female race walkers showed a significant decrease in 10-km race times after a 5.5-week LCD [24]. In a two-arm randomized controlled trial, seven competitive male runners that followed a six-week ketogenic diet had no significant change in their 5-km time trial at days 4 and 42, nor did they have a difference in time to exhaustion after six weeks of consuming an LCD [25]. In another randomized cross-over trial, 14 recreational male athletes had no significant change in work output on a 90-minute cycle ergometry test after two weeks on a low-carbohydrate, high-fat diet [26]. In a randomized, repeated measures crossover study, eight trained male endurance athletes were tested in a run-to-exhaustion time trial following a 31-day ketogenic diet [27]. When the respiratory exchange rate (RER) was >1.0, indicative of anaerobic conditions and a greater dependence on carbohydrate metabolism, there was a statistically significant decrease in the run time to exhaustion. However, for an RER <1.0, there was no statistically significant change. In addition, the athletes who completed the ketogenic diet had impaired exercise efficiency, which was more pronounced at >70% VO_2_max. In a randomized crossover trial, eight recreational male swimmers had no significant change in swimming economy at 50%, 60%, and 70% VO_2_max after a three-day LCD [28]. In a randomized controlled trial, six well-trained competitive cyclists found a significant decrease in time-to-exhaustion cycling trials at 10°C and 30°C after a three-day LCD [29]. In a comparative study, 22 professional male soccer players significantly decreased the total distance covered in a 90-minute soccer game after a four-day LCD [30]. Together, these results suggest inconsistent effects of an LCD on aerobic performance, likely due to differences in the characteristics of individuals in the samples, specifics of the dietary intervention, and athletic parameters measured.

**Table 1:** Studies on the effects of a low-carbohydrate or ketogenic diet on aerobic exercise performance tests Note that studies are ordered by increasing the length of dietary intervention. * p<0.05, † 10-km race times estimated from graphs β-HB: beta-hydroxybutyrate, g: grams, HCD: high-carbohydrate diet, KD: ketogenic diet, kJ: kilojoules, LCD: low-carbohydrate diet, m: meters, min: minutes, mL O2/kCal: milliliters of oxygen/kilocalorie, n.s.: not significant, RCT: randomized controlled trial, RER: respiratory exchange rate, RTE: run to exhaustion trial time, s: seconds, WD: Western diet, y/o: years old

Study	Study design and participants	Diet intervention	Performance before LCD	Performance after LCD	Compliance
Bestard et al., 2020 [28]	Randomized-crossover trial; 8 healthy, recreational swimmers, swimming >3 km/week, 34.6 ± 9.4 y/o	3-day LCD: 67% fats, 18% protein, and 16% CH_2_O; 3-day HCD: 70% CH_2_O	HCD day 3: 50% Vmax swimming Economy 654 J/m, 60% Vmax swimming economy 679 J/m, 70% Vmax swimming economy 755 J/m	LCD day 3: 50% Vmax swimming economy 649 J/m (n.s), 60% Vmax swimming economy 654 J/m (n.s.), 70% Vmax swimming economy 711 J/m (n.s)	Self-reported food and drink intake
Pitsiladis and Maughan 1999 [29]	RCT w/2 arms (LCD and HCD); 6 well-trained competitive cyclists, 28 ± 8 y/o	3-day LCD: 65% fats, 26% protein, and 9% CH_2_O; 3-day HCD: >80% CH_2_O	HCD day 3: cycling time to exhaustion at 10°C: 158.2 min; 30°C: 53.2 min	LCD day 3 cycling time to exhaustion at 10°C: 89.2 min*; 30°C: 44 min*	Not reported, but subjects were supplied with diet sheets containing the type and weight of the individual food items to be consumed daily
Souglis et al., 2013 [30]	Comparative study; 22 professional male soccer players 24 ± 0.7 y/o on one team, 24.5 ± 0.8 y/o on other team	4-day LCD: 3g/kg body mass; 4-day HCD: 8g/kg body mass	HCD day 4: total distance covered during 90 min soccer game in 1st half: 4,815 ± 51 m; 2nd half: 4,565 ± 50 m; mean total: 9,380 ± 98 m	LCD day 4: total distance covered during 90 min soccer game in 1st half: 4,226 ± 50 m*; 2nd half: 3,851 ± 61 m*; mean total: 8,077 ± 109 m*	Not reported. Both diets were isocaloric and designed by a registered dietitian
Baart et al., 2023 [22]	Randomized-sequence crossover trial; 14 males, 22-44 y/o, recreational athletes that trained >4 hours/week	14-day LCD: 75% fats, <10% carbohydrates	Exercise efficiency 17.6 ± 1.9 %; exercise economy 1191 ± 138 mL O_2_/kCal	Exercise efficiency 18.2 ± 1.2 % (n.s.), exercise economy 1143 ± 75 mL O_2_/kCal (n.s.)	Self-reported food weight pre-consumption and recorded any deviations from the prescribed diet
Terink et al., 2021 [26]	Randomized, cross-over trial; 14 males, 18-45 y/o, recreational athletes who regularly trained >4 hours/week	14-day LCD: 75% fats, <10% carbohydrates	90 min cycle ergometry work (2 days into LCD) 939 ± 163 kJ	90 min cycle ergometry work (14 days into LCD) 1003 ± 129 kJ (n.s.)	Serum ketones measured before and 1 hour after exercise performance tests
Burke et al., 2017 [23]	Clinical trial w/pretest and posttest; 21 professional male race walkers, LCD mean 28.3 y/o, HC mean 25.4 y/o	21-day low-CH_2_O diet: 78% fats, 17% protein, and 3.5% CH_2_O	10-km race time ~ 43.5 min†	10-km race time ~ 44.5 min (n.s.)†	Individualized meal plans; food weight recorded at each meal; serum β-HB measured weekly
Paoli et al., 2021 [20]	RCT w/2 arms (KD or WD); 16 males, 25.5 ± 2.8 y/o, semi-professional soccer players	30-day LCD: 70% fats, 25-30% protein, and 10% carbohydrates	Yo-yo performance test: 880.4 ± 244.8 m	Yo-yo performance test: 1123 ± 266.8 m*	Urinary acidity measured weekly; serum ketones measured weekly
Shaw et al., 2019 [27]	Randomized, repeated-measures, crossover study; 8 males, 29.6 ± 5.1 y/o, trained endurance athletes	31-day KD: 75-80% fats, 15-20% protein, <50g carbohydrates	RTE when RER >1.0 at VO_2_max 237 ± 31 min, RTE when RER <1.0 at VO_2_max 241 ± 27 min	RTE when RER >1.0 at VO_2_max 174 ± 24 min*, RTE when RER <1.0 at VO_2_max 265 ± 21 min (n.s.)	Urinary ketones measured daily; serum β-HB on days 3, 7, 14, 21, and 28 before breakfast and exercise
Burke et al., 2020 [24]	Clinical trial w/pre-test and post-test; 26 professional male and female race walkers, LCD mean 29.0 y/o, HCD mean 25.5 y/o	5.5-week LCD: 75-80% fats, 20% protein, and <50g carbohydrates daily	10-km IAAF race points (converted race times) 1034 points	10-km IAAF race points (converted race times) 976 points*	Individualized meal plans, food weight recorded at each meal, serum β-HB measured weekly
Prins et al., 2019 [25]	RCT w/2 arms (LCD or HCD); 7 competitive male recreational distance runners, 35.6 ± 8.4 y/o with 15.1 ± 7.1 years running experience	6-week LCD: 80% fats, 15-20% protein, and <50g carbohydrates	Pre-diet 5-km time trial time to exhaustion 844.3 ± 87.3 s; 5-km time trial on day 4: 1231 ± 137 s	Day 39 5-km time trial time to exhaustion 818.6 ± 114.5 s (n.s.); 5-km time trial on day 42: 1211 ± 152 s (n.s)	Serum ketones daily (LCD) 3-day, weighed food records (LCD and HCD)

Anaerobic Exercise Performance

Results of studies of ketogenic diets of varying lengths on anaerobic exercise performance tests are summarized in Table 2. In a randomized sequence crossover trial of a four-day ketogenic diet with exercise-trained participants, there was a statistically significant increase in anaerobic capacity as measured by the Wingate test [19]. However, in studies where the ketogenic diet was longer, anaerobic performance either stayed the same or decreased [31,32]. In a clinical trial with a four-week LCD, there was a notable drop in anaerobic performance in basketball players, with significantly decreased performance on the Wingate test [31]. This contrasts with a randomized clinical trial using a 10-week LCD with trained college-aged men, in which researchers found that subjects’ Wingate performance test results did not significantly change [32]. Therefore, the effects of a ketogenic diet on anaerobic exercise performance, as assessed by the Wingate test, also do not follow a single trend; performance may increase or decrease, depending upon the length or specifics of the dietary intervention, the population sampled, or additional factors. More research is needed to determine sources of variability for the observed outcomes.

**Table 2:** Studies on the effects of a low-carbohydrate or ketogenic diet on anaerobic exercise performance tests Note that studies are ordered by increasing the length of dietary intervention. * p<0.05 β-HB: beta-hydroxybutyrate, J/kg: joules/kilogram, KD: ketogenic diet, LCD: low-carbohydrate diet, n.s.: not significant, RCT: randomized controlled trial, W: watts, WD: Western diet, y/o: years old

Study	Study design and participants	Diet intervention	Performance before LCD	Performance after LCD	Compliance
Wroble et al., 2019 [19]	Randomized-sequence crossover trial: 6 males and 10 females, 23 ± 1 y/o, exercise-trained (≥ 3 moderate to high-intensity exercise sessions/week for ≥30 min/session over the past ≥ 6 months)	4-day LCD: 69.7% fats, 22.2% protein, and 9.3% carbohydrates	Wingate test power: 564 ± 50 W	Wingate test power: 598 ± 51 W*	Urinary acidity measured daily
Michalczyk et al., 2019 [31]	Clinical trial w/pretest and posttest; 15 males, 23.5 ± 2.2 y/o, basketball players w/5 years of training	4-week LCD: 59% fats, 31% protein, and 10% carbohydrates	Wingate test total work: 301.17 ± 12.42 J/kg	Wingate test total work: 266.69 ± 6.46 J/kg*	β-HB measured weekly. Controlled meals in the cafeteria
Wilson et al., 2020 [32]	RCT w/ 2 arms (KD or WD); 25 college men, 18-30 y/o, 5.5 ± 3.8 years of training	10-week LCD: 75% fats, 20% protein, and 5% carbohydrates	Wingate test power: 849.78 ± 182.72 W	Wingate test power: 834.4 ± 177.1 W (n.s.)	β-HB measured weekly

Upper and Lower Body Strength Performance

Studies of upper and lower body strength after a ketogenic diet are summarized in Table 3. A one-repetition maximum weighted bench press was used to measure strength in the upper body. In contrast, a one-repetition maximum weighted squat or counter-movement jump (CMJ) test was utilized to measure lower body strength [32,33]. To assess upper body strength, a randomized, parallel arm, controlled prospective study tested the one-repetition bench press maximum load of 21 strength-trained women before and after an eight-week LCD; no significant change was observed [33]. In another two-arm randomized clinical trial, 20 experienced resistance-trained men underwent an eight-week ketogenic diet; again, no substantial change in their one-repetition bench press maximum load [31]. However, a different two-arm randomized clinical trial found a statistically significant increase in bench press after a 10-week ketogenic diet in 25 resistance-trained college-aged men [32]. Another randomized controlled parallel study, which utilized an eight-week ketogenic diet and included 19 male athletes, also observed that the participants significantly increased their bench press [21]. For lower body strength, a randomized, parallel arm, controlled prospective study tested the one-repetition maximum squat load of 21 strength-trained women after an eight-week LCD; a significant increase was found in both one-repetition maximum squat load and CMJ [33]. Similarly, a two-arm randomized clinical trial found a statistically significant increase in one repetition maximum squat load after a 10-week ketogenic diet in 25 trained college-aged men [32]. Another randomized controlled parallel study had similar results, in which 19 male athletes significantly increased their one-repetition maximum squat load after an eight-week ketogenic diet [21]. However, in a two-arm randomized clinical trial, 20 experienced resistance-trained men who underwent an eight-week ketogenic diet had no significant change in their one-repetition maximum squat load [34].

To assess the effects of a six-week ketogenic diet on total upper and lower body strength, a single-arm within-subjects clinical trial examined the effects on total workout repetitions per week and maximum volume load, the multiplication of weight used by the set and number of repetitions, in 13 resistance-trained individuals. There were statistically significant increases of 11.4% in weekly repetitions and 7.6% in volume load [35]. In a randomized crossover study, 14 trained powerlifters self-selected a lift of choice and underwent a three-month ketogenic diet. There was no significant change in their self-selected lift’s one-repetition maximum load before and after a three-month ketogenic diet [36]. The studies on upper and lower body strength indicate the potential for some performance benefit on an LCD [21,32,33,35], although this is not uniformly the case [34,36].

**Table 3:** Studies on the effects of a low-carbohydrate or ketogenic diet on upper and lower body strength Note that studies are ordered by increasing the length of dietary intervention. * p<0.05 β-HB: beta-hydroxybutyrate, CMJ: counter-movement jump, g: grams, KD: ketogenic diet, kg: kilograms, LCD: low-carbohydrate diet, NKD: non-ketogenic diet, n.s.: not significant, RCT: randomized controlled trial, reps: repetitions, RM: repetition maximum weight, WD: Western diet, y/o: years old

Study	Study design and participants	Diet intervention	Performance before LCD	Performance after LCD	Compliance
Vargas-Molina et al., 2024 [35]	Single-arm, non-randomized, own-controlled clinical trial; 14 resistance-trained individuals: 3 female, 11 male; mean 30.1 y/o	6-week LCD: 2g protein/kg body weight, 30-40g carbohydrates	Week 0: 170.5 reps/week, volume load 325.6 ± 49.7 sets/week	Week 6: 190.0 reps/week*, volume load 350.4 ± 54.7 sets/week*	Urinary ketones measured weekly
Paoli et al., 2021 [21]	Randomized controlled parallel study; 19 male athletes, 27.4 ± 10.5 y/o	8-week KD: 68% fat, 25% protein, 5% carbohydrates	Week 0: bench press 1 RM: 129.78 ± 20.98 kg, squat 1 RM: 181.33 ± 36.52 kg	Week 8: bench press 1 RM: 134.44 ± 17.14 kg*, squat 1 RM: 187.78 ± 37.41 kg*	Serum β-HB measured weekly
Vargas-Molina et al., 2020 [33]	Randomized, parallel arm, controlled, prospective study; 21 strength-trained women, 27.6 ± 4.0 y/o	8-week LCD: 65% fats, 26% protein, and <10% carbohydrates	Baseline: bench press 1 RM: 41.5 + 8.4 kg, squat 1 RM: 68.5 + 11.2 kg, CMJ: 20.8 + 2.7 cm	Week 8: bench press 1 RM: 43.0 + 7.7 kg (n.s), squat 1 RM: 74.1 + 12.3 kg*, CMJ: 22.4 + 3.3 cm*	Urinary ketones measured weekly
Vidic et al., 2021 [34]	RCT w, 2 arms (KD or NKD); 20 noncompetitive experienced resistance-trained men, 42.7 ± 1.5 y/o	8-week LCD: 75% fats, 20% protein, 5% carbohydrates	Week 0: bench press 1 RM: 103 ± 6.09 kg, squat 1 RM: 122.5 ± 6.3 kg	Week 8: bench press 1 RM: 102.2 ± 5.5 kg (n.s.), squat 1 RM 121.3 ± 7.0 kg (n.s.)	Serum β-HB measured weekly
Wilson et al., 2020 [32]	RCT w/ 2 arms (KD or WD); 25 college men, 18-30 y/o, 5.5 ± 3.8 years of training experience	13 subjects ate a 10-week LCD: 75% fats, 20% protein, 5% carbohydrates	Week 0: bench press 1 RM: 252.7 ± 44.8 kg, squat 1 RM: 287.31 ± 55.1 kg	Week 10: bench press 1 RM: 261.2 ± 44.8 kg*, squat 1 RM :303.1 ± 59.4 kg*	Serum β-HB measured weekly
Greene et al., 2018 [36]	Randomized, crossover study; 14 intermediate-to-elite powerlifters and Olympic weightlifters, 35 ± 11 y/o	3-month KD: <10% calories from carbohydrates	1 RM strength of a self-selected lift: 132 kg	1 RM strength of a self-selected lift: 135 kg (n.s.)	Serum β-HB and glucose measured weekly

Results by length of dietary intervention

Four studies with a diet intervention lasted less than one week. In one randomized crossover trial, the swimming economy of eight healthy recreational swimmers was examined after three days of an LCD or HCD. The tests were performed at 50%, 60%, and 70% Vmax; no significant changes in swimming economy were found between the low- and high-carbohydrate groups [28]. A randomized controlled trial with two arms compared cycling time-to-exhaustion in six competitive cyclists after a three-day LCD or HCD at 10°C and 30°C. There was a statistically significant decrease in cycling time-to-exhaustion in the low-carbohydrate group compared to the high-carbohydrate group at both temperatures [29]. A comparative study tested the total distance covered in a soccer game with 22 professional male soccer players who consumed a four-day LCD or HCD. There was a statistically significant decrease in the total distance covered in the low-carbohydrate group compared to the high-carbohydrate group in the first half of the game, the second half, and the total game [30]. A randomized-sequence crossover trial also utilized a four-day LCD, in which 16 exercise-trained men and women were assessed with the Wingate test. There was a statistically significant increase in the Wingate power after the short dietary intervention, both in men and women [19]. While these studies with an LCD of less than one week generally do not suggest a benefit for this length of intervention, except for power [19], at least one study indicates a possible detriment in endurance [30].

Six studies with a diet intervention lasted between 14 and 31 days. A randomized-sequence crossover trial that tested exercise efficiency and economy in 14 recreational male athletes after a 14-day LCD found no significant change in either parameter [22]. A different randomized crossover trial tested 90 minutes of cycle ergometry work in 14 recreational male athletes after a 14-day LCD; no statistically significant change was found in this parameter [26]. A clinical trial that assessed 10-kilometer race times in 21 professional male race walkers after a 21-day LCD found no significant change in race times [23]. However, a clinical trial that used a four-week LCD found a statistically significant decrease in the Wingate test total power in 15 well-trained male basketball athletes [31]. A two-arm randomized controlled trial with 16 semi-professional male soccer players who followed a 30-day LCD found a statistically significant increase in the Yo-Yo intermittent recovery test that assesses aerobic performance [20]. A randomized crossover study was used to test run-to-exhaustion trial times after a 31-day ketogenic diet. In this study with eight trained male endurance athletes, there was a statistically significant decrease in the run-to-exhaustion trial time when the RER was >1.0 but no significant change when the RER was <1.0 [27]. Together, studies on LCD of two weeks to a month in duration were generally found to provide no benefit to athletes, aside from an increase in aerobic performance in one study [20], with two studies finding a detriment to power [31] or endurance within high RER conditions [27].

Eight studies with a diet intervention lasted between 5.5 and 12 weeks. A clinical trial tested 10-km race points (IAAF points) in 26 professional male and female race walkers after a 5.5-week LCD; statistically significant decreased race times were reported [24]. A single-arm within-subjects clinical trial tested repetitions per week and volume load in 14 resistance-trained individuals after a six-week LCD; statistically significant increases in both parameters were reported [34]. A two-arm randomized controlled trial involved seven competitive male recreational distance runners who followed a six-week LCD. No statistically significant change was found in either time to exhaustion or time to complete a 5 km run on day 39 of the diet compared to the baseline [25]. A randomized, parallel-arm, controlled, prospective study tested the bench press, squat, and countermovement jump in 21 strength-trained women on an eight-week LCD. While no significant change was reported in the bench press, there was a significant increase in the squat and countermovement jump test after dietary intervention [33]. In a two-arm randomized controlled trial, bench press and squat were tested on 29 experienced resistance-trained men following an eight-week ketogenic diet, with no significant change reported in either parameter [36]. A randomized controlled parallel study tested bench press and squat one-repetition maximum in a study with 19 male athletes following an eight-week ketogenic diet; here, a statistically significant increase was found in both the bench press and squat tests [21]. Similarly, a two-arm randomized controlled trial that involved 25 trained men following a 10-week ketogenic diet found a statistically significant increase in bench press and squat maximum; however, no difference was found in Wingate power [32]. Lastly, a randomized, crossover study tested a one-repetition maximum of a self-selected lift in 14 intermediate-to-elite level powerlifters and Olympic weightlifters following a three-month ketogenic diet; no significant differences were reported [35]. These studies, taken together, suggest that an LCD may have some benefit for improving measures of strength if the diet is adhered to for one to several months.

Discussion of the impact of a low-carbohydrate diet on competitive athlete performance

Aerobic Performance

There are multiple measures of aerobic fitness performance, including the Yo-Yo intermittent recovery test, time to exhaustion in endurance activities, and exercise economy, which is the volume of oxygen required to move at a given power output or speed (a predictor of endurance performance) [37-39]. Aerobic performance relies largely on the efficiency of ATP production from various energy sources, including carbohydrates, amino acids, and fatty acids [40]. There were no statistically significant differences in aerobic performance for 10-kilometer race times in studies with a 21-day or 5.5-week LCD [23,24]. Furthermore, there were no statistically significant changes in a 5-km time to exhaustion time or a 5-km time trial following a six-week LCD [25], nor was there a change in a 90-minute cycle ergometry work test after a 14-day LCD [26]. Exercise economy and efficiency directly translate to exercise performance [38,39], but these did not significantly change after a 14-day LCD. Together, most studies suggest that a ketogenic diet does not impair or help aerobic performance but also does not improve aerobic performance. As such, an LCD could be an alternative to a traditional high-carbohydrate diet if desired for other goals, such as weight loss or glycemic control.

Although there was no statistically significant change in aerobic exercise performance for a run to exhaustion time with an RER <1.0, there was a statistically significant decrease when an RER >1.0 [27]. When RER <1.0, proteins and fatty acids are predominately used to fuel exercise, whereas carbohydrates are used as the primary fuel source during anaerobic conditions when the RER >1.0 [41,42]. From these studies, there is evidence that suggests that anaerobic performance can be hindered by an LCD (RER >1.0). For athletes who want to utilize an LCD, measuring the RER may be reasonable to use as a predictor of whether performance will decrease. In contrast, there was a statistically significant increase in the Yo-Yo performance test after a 30-day LCD, suggesting a potential increase in aerobic performance when following an LCD [20]. Overall, athletes who rely primarily on aerobic performance should be aware that an LCD will likely neither hinder nor help them concerning athletic performance. However, anaerobic performance may decline on this type of diet [27].

Regarding performance on an LCD when specifically compared with that on a high-carbohydrate diet, a randomized crossover study in seven male endurance runners found better maintenance of aerobic performance measured as an 8-kilometer or 16-kilometer running time trial when following an 11-day high-carbohydrate diet compared to baseline [43]. A previous meta-analysis analyzed twenty published trials that assessed endurance performance based on diet and found that the subjects following a high-carbohydrate diet exercised longer to exhaustion. However, trained athletes were less likely to gain performance benefits from the high-carbohydrate diet [44]. Furthermore, a crossover study analyzed the effects of a high-carbohydrate diet on 10-kilometer run times in young runners and found that the high-carbohydrate diet was correlated with better running performance and running speed [45]. A randomized trial of placing professional cycling athletes on a seven-day high-carbohydrate diet saw a faster time in a 25-kilometer cycling trial in athletes with a lower aerobic capacity [46]. Together with the results of studies reviewed here, these findings point to a high-carbohydrate diet having better outcomes for aerobic performance than an LCD.

Anaerobic Performance

Anaerobic performance tests included the Wingate test, a 30-second all-out ergometry test in which an individual pedals against resistance determined by a certain percentage of their body weight. The Wingate test is not limited to measuring anaerobic capacity but can also measure lower body peak power [47,48]. The first five seconds of the Wingate test usually contain the peak power output measurement. In comparison, the full 30 seconds contain an estimate of anaerobic capacity, determined by the availability of ATP-phosphocreatine reserves and glycolytic metabolism [49]. Three of the studies used the Wingate test to measure the effects of an LCD on anaerobic performance [19,31,32]. Performance in athletes varied, ranging from a statistically significant increase [19] to a decrease [31] to no change in anaerobic performance [32]. A key difference between these studies was the length of the diet, with the increased performance occurring with a short four-day LCD [19], the decreased performance occurring with a four-week LCD [28], and no change in performance with a 10-week LCD [32]. Therefore, perhaps anaerobic performance depends on the length of the LCD; however, additional studies are needed to understand this relationship better. High-carbohydrate diets, in comparison, also did not result in significant effects on time to exhaustion for high-intensity activity by trained cyclists after two weeks of an HC diet [50] nor on high-intensity interval swim training for collegiate swimmers who followed a nine-day HC diet [51].

Upper Body Strength Performance

The bench press is a compound lift that targets many of the upper body's muscles and is a reliable indicator of upper body strength [52]. In this systematic review, two studies showed no significant change in the one-repetition maximum bench press weight; both studies utilized an eight-week LCD [33,36]. However, two other studies showed a significant increase in the one-repetition maximum bench press weight after a 10-week LCD or an eight-week LCD [21,32]. Therefore, upper body strength is likely not decreased by an LCD. However, as many athletes aim to gain muscle, an LCD might not be optimal for upper body strength gains, based on the discrepant findings on the bench press maximum weight. Therefore, athletes who want to utilize an LCD for upper body muscle strength gains should do so with caution, as there is a chance for stagnancy in their maximum bench press weight.

Lower Body Strength Performance

The CMJ test can monitor sports performance, assessing lower body strength and mechanical performance [53]. Only one study measured the countermovement jump test and found a statistically significant increase in the test after an eight-week LCD [33]. The squat is a compound lift that targets many of the muscles of the lower body and is a reliable indicator of lower body strength [54]. In this systematic review, three studies found an increase in the maximum one-repetition squat weight; two used an eight-week LCD, and one used a 10-week LCD [21,32,33]. However, one study showed no statistically significant change in one-repetition maximum squat weight after an eight-week LCD [36]. If athletes want to gain lower body muscle strength, then the LCD diet seems to favor gaining muscle strength in the lower body.

Strengths of studies

All the studies have described specific macronutrient dietary compositions, breaking down the proportion of carbohydrates, proteins, and fats, as shown in Tables 1-3. Having specific macronutrient ratios that make up the LCD ensures that the diet can be properly replicated by athletes seeking the effects reported. Furthermore, specifically listing the proportion of protein in the diet is important, as protein content may have confounding effects with an LCD; protein causes glycogenesis from amino acids within the first few days of an LCD [55], and protein is necessary for muscle hypertrophy [56] and may therefore particularly impact studies that analyze the effects of an LCD on upper and lower body strength performance.

A particular strength of many of the studies is adequate time for dietary intervention to ensure ketosis. Studies have placed the average time to achieve ketosis when utilizing an LCD from two to four days, although some individuals may take longer [57,58]. Four of the 18 studies reviewed here utilized a three-to-four-day LCD; all other studies used an LCD that lasted two weeks or longer. Therefore, ketosis was achieved, as supported by urinary and serum ketone levels.

Limitations

Many studies compared a low-carbohydrate group to a control group, in which the intervention was either a high-carbohydrate or a typical Western diet. However, a more powerful comparison would be a within-subjects pre- and post-intervention study to investigate the effects of an LCD on the same population of trained individuals. Having the same population greatly reduces the random error associated with between-subject comparisons. A study design with two counterbalanced arms and a washout period between diets would be appropriate.

Some studies assessed participants’ adherence to the diet intervention via participant self-reporting [22,28], or adherence was not reported [30]. Strict adherence to an LCD should be monitored quantitatively to ensure this isn’t a confounding factor. For example, blood or urine ketones could be measured every few days to ensure ketosis and adherence to the LCD rather than relying on participant recall of dietary composition and serving sizes [22,28,30].

Several studies were judged to have a high risk of bias, which was related to lack of allocation concealment [7,23,24,33], lack of blinding of participants and personnel [24], lack of random assignment [7,33], or lack of clarity on one or two of these domains [7,29-31,34]. In conjunction with the limited number of studies conducted to date, inconsistencies in findings, small sample sizes, and limited statistical power, this highlights the deficiency of data needed to make a strong recommendation on using an LCD to improve athlete performance. Additionally, well-designed studies that address the impact of dietary macronutrient composition on measures of athletic performance are needed to guide athletes better.

Future directions

More research is needed on the effects of different diet types on anaerobic, aerobic, upper body strength, and lower body strength performance to provide better recommendations for athletes.

Recommendations for athletes

Current evidence is not adequate enough to support using an LCD diet for athletic performance, particularly over a high-carbohydrate or high-protein diet. For athletes who want to follow an LCD diet, guidelines are based on their primary goal: anaerobic performance, aerobic performance, and upper and lower body strength performance. For upper and lower body strength, for athletes who want to maintain muscle mass, and for upper and lower body strength performance, an LCD does not appear to be detrimental but has not been shown to provide consistent benefits. For anaerobic activity, the effects of an LCD on anaerobic performance are inconsistent but may decrease performance. An LCD has not been shown to provide consistent benefits. For aerobic activity, the effects of an LCD on aerobic performance are inconsistent and generally have not been shown to provide benefits.

## Conclusions

While an LCD has been popularized for potential weight loss benefits, athletes do not use it widely. This is likely due to the restriction of an essential source of energy for exercising muscles. An LCD does not provide consistent benefits concerning anaerobic or aerobic performance in assessments such as the Wingate test, run-to-exhaustion trials, Yo-Yo intermittent recovery test, or 10-kilometer race times; it may even reduce performance. An LCD may provide limited benefits for upper and lower body performance tests such as the bench press, squat, and CMJ test. While further studies may shed more light on the impact of an LCD on athletic performance, the existing evidence does not support this being a diet of choice for athletes.
